# β-glucan mitigates necrotizing enterocolitis-induced long-term cognitive impairment by remodeling gut microbiota and restoring tryptophan metabolite IAA-mediated gut–brain signaling

**DOI:** 10.1080/19490976.2026.2756699

**Published:** 2026-11-07

**Authors:** Xingdao Zhang, Haiyan Cao, Yuni Zhang, Yuan Shi

**Affiliations:** a Department of Neonatology, National Clinical Research Center for Child Health and Disorders, Ministry of Education Key Laboratory of Child Development and Disorders, Children’s Hospital of Chongqing Medical University, Chongqing, China; b Chongqing Key Laboratory of Structural Birth Defect and Reconstruction, Chongqing, China; c Bishan Maternal and Child Health Care Hospital, Chongqing, China

**Keywords:** β‑glucan, cognitive impairment, necrotizing enterocolitis, gut microbiota, tryptophan metabolism, FMT, IAA

## Abstract

Necrotizing enterocolitis (NEC) is a life-threatening gastrointestinal disorder prevalent in preterm infants, and most NEC survivors suffer from persistent cognitive deficits and neurodevelopmental disorders, with no targeted therapeutic approaches available to date. *β*-glucan has been documented to regulate gut microbiota, suppress inflammation, and exert neuroprotective effects, yet its efficacy and molecular mechanisms against NEC-associated cognitive dysfunction remain unclear. In this study, we established a mouse NEC model with *β*-glucan intervention to explore its gut–brain axis protective effects. Behavioral assessments verified that *β*-glucan significantly rescued NEC-induced cognitive impairments, with comparable therapeutic effects between low and high doses. Multi-omics analyses demonstrated that *β*-glucan effectively reversed NEC-triggered gut microbiota dysbiosis and restored disrupted tryptophan metabolism, notably elevating the level of indole-3-acetic acid (IAA). Fecal microbiota transplantation and exogenous IAA supplementation recapitulated the neuroprotective phenotypes of *β*-glucan, which inhibited excessive hippocampal microglial activation and neuroinflammation, and repaired impaired synaptic ultrastructure and plasticity. Further mechanistic validation confirmed that IAA mediated neuroprotection via activating the AHR/MAP2K3/p38 signaling cascade. Collectively, *β*-glucan alleviates NEC-related cognitive injury through modulating the gut microbiota–tryptophan metabolism–IAA axis and subsequent AHR/MAP2K3/p38 signaling activation. This study elucidates a novel gut‒brain axis regulatory mechanism of *β*-glucan, providing reliable preclinical evidence for the clinical prevention and treatment of long-term neurodevelopmental complications in NEC survivors.

## Introduction

Necrotizing enterocolitis (NEC) is one of the most critical gastrointestinal emergencies in preterm infants, pathologically characterized by acute intestinal necrosis and systemic inflammatory storm.^1^ ^,^ ^2^ Its high mortality rate and long-term neurological sequelae have become a major challenge in the field of neonatal critical care medicine. Although innovations in neonatal intensive care technology have markedly improved the survival rate of infants with NEC, approximately 30%–50% of survivors suffer from persistent neurodevelopmental abnormalities, including cognitive retardation, learning and memory impairment, and cerebral palsy.^3-6^ These complications severely impair the long-term quality of life of affected children and impose a heavy burden on families and society. Current clinical interventions are limited to symptomatic supportive treatments for acute intestinal injury, such as anti-infection and nutritional support.^7^ There are still no targeted pharmacological strategies to reverse NEC-induced secondary brain damage.^8^ Therefore, elucidating the core pathogenesis of NEC-associated neurocognitive disorders and screening safe and effective natural therapeutic agents have become urgent clinical and scientific issues.

The gut–brain axis serves as a vital regulatory network maintaining functional communication between the intestine and the central nervous system, and acts as a core pathway mediating remote neurological injury induced by intestinal diseases.^9-15^ Intestinal barrier disruption, gut microbiota dysbiosis, and release of intestinal-derived inflammatory factors triggered by NEC can penetrate the blood–brain barrier via the “microbiota–metabolite–neuroinflammation” axis, including humoral transmission and vagus nerve conduction. These pathological events induce excessive activation of hippocampal microglia, trigger a neuroinflammatory cascade, and ultimately lead to impaired synaptic plasticity and cognitive decline.^7^ ^,^ ^16^ This pathological mechanism suggests that targeted regulation of gut–brain axis homeostasis, restoration of intestinal microecology, and supplementation of key microbial active metabolites may represent a promising breakthrough for intervening in long-term cognitive sequelae after NEC.

*β*-glucan is a type of bioactive polysaccharide naturally derived from yeast, Lentinus edodes, oats, and other sources. Owing to its favorable biosafety, it has been widely applied as a dietary supplement.^17^ Accumulating studies have confirmed that *β*-glucan exhibits multiple pharmacological activities, including anti-tumor,^18^ anti-obesity,^19^ anti-inflammatory,^20^ gut microbiota regulation,^21-23^ and immunomodulatory effects.^24^ Notably, substantial evidence has validated its prominent neuroprotective properties.^25-27^ In disease models of Alzheimer’s disease,^28^ ^,^ ^29^ Parkinson’s disease^30^ and age-related cognitive decline,^31^ *β*-glucan ameliorates cognitive deficits by remodeling gut microbiota composition, suppressing central neuroinflammation, and maintaining synaptic integrity. These findings break the traditional perception of *β*-glucan merely as an intestinal protectant and highlight its potential for cross-organ neuroprotection. However, existing studies on *β*-glucan in NEC are mainly confined to its protective effect on the intestinal mucosal barrier. Whether *β*-glucan can be extended to intervene in long-term neurobehavioral abnormalities, and whether its function relies on gut microbiota-mediated gut–brain axis regulation, remains to be systematically verified, representing an important research gap to be filled in this field.

Based on the above background, the present study aimed to clarify the therapeutic effect of *β*-glucan on long-term cognitive impairment induced by NEC. Here, long‑term follow‑up was defined as observation until postnatal week 4 (P28, early‑juvenile stage in mice), to explore sustained gut–brain‑axis dysfunction following neonatal NEC injury. With gut microbiota regulation as the core entry point, we further systematically elucidated the intrinsic mechanism underlying its cross-organ neuroprotection. The findings of this study are expected to provide novel mechanistic insights, potential therapeutic targets, and preclinical experimental evidence for the prevention and treatment of long-term neurodevelopmental complications in NEC survivors.

## Methods

### Establishment of the NEC Model and long-term survival protocol

Seven‑day‑old (P7) C57BL/6J mouse pups were randomly selected from multiple independent litters and allocated to control and NEC groups. No sex‑based matching or stratification was performed during randomization. The NEC model was induced from P7 to P11 by gavaging the pups every 3–4 h with a hyperosmolar formula (Similac Advance and Esbilac, 3.3:2; 30–50 μL/10 g body weight), combined with hypoxia (100% N₂, 90 s) and cold stress (4 °C, 10 min) twice daily. Control pups remained with their mothers for normal lactation. To assess long‑term neurocognitive outcomes, surviving neonatal pups with necrotizing enterocolitis were fostered by healthy surrogate dams, weaned on postnatal day 21, and housed in a standard specific‑pathogen‑free (SPF) environment until postnatal week 4 (postnatal day 28) for testing and sample collection.

It is worth noting that this study did not sacrifice animals during the acute phase of model establishment for histological verification of necrotizing enterocolitis in intestinal tissues. Therefore, mice enrolled in the long‑term observation cohort could not obtain pathologically confirmed diagnoses of necrotizing enterocolitis at the individual‑animal level. Only mice that survived the acute modeling phase of necrotizing enterocolitis reached the long‑term observation endpoint at postnatal week 4 (P28).

Mice at postnatal day 28 (P28, 4‑week‑old, C57BL/6J) were anesthetized by inhalation of isoflurane before transcardial perfusion. Isoflurane vaporizer settings: 3.5%–4.0% isoflurane mixed with oxygen (0.8–1.0 L/min) for induction; 1.5%–2.0% isoflurane was applied via nose‑cone for maintenance of surgical‑grade anesthesia during tissue collection. Terminal euthanasia was performed via carbon dioxide (CO₂) inhalation, and cervical dislocation was applied as a secondary physical method to ensure death. All anesthesia and euthanasia procedures complied with institutional animal care guidelines and the American Veterinary Medical Association (AVMA) guidelines for laboratory animals.

The animal study has been approved by the Ethics Committee of the Children’s Hospital of Chongqing Medical University (No. CHCMU-IACUC20251011011).

### Detection and sampling time points

As shown in Figures 1A and 5A, treatments with vehicle, β‑glucan, indole‑3‑acetic acid (IAA), and CH223191 were all initiated on postnatal day 12 of neonatal mice, after completion of the necrotizing enterocolitis (NEC) modeling procedure. As shown in Figure 3A, mice in the NEC group and fecal microbiota transplantation (FMT) group required antibiotic treatment on day 12 prior to receiving vehicle and fecal supernatant interventions.

Detection and sampling time points: At postnatal week 4 (P28), mice were randomly selected from each group for behavioral assessment as well as fecal and hippocampal tissue sampling. Mice subjected to behavioral tests were not sacrificed for hippocampal tissue collection for subsequent analysis, so as to exclude potential confounding interference of behavioral tests on molecular and histological detection results.

### Behavioral testing

To evaluate the long-term neurocognitive impacts of NEC, PW4 mice underwent behavioral testing in a sound-attenuated, environmentally controlled room. All activities were continuously recorded and analyzed using the ANY-maze tracking system. Mice assigned to behavioral assessments were derived from multiple independent litters; pups originating from different dams were distributed across all experimental groups.

#### Open field test (OFT)

Spontaneous locomotion and anxiety-like behaviors were assessed using an open-top square arena (40 × 40 × 40 cm). Mice were placed in the center and allowed to freely explore for 5 min. Total distance traveled and time spent in the central zone (20 × 20  cm) were quantified. The apparatus was wiped with 75% ethanol between trials.

#### Novel object recognition (NOR) test

Non-spatial working memory was evaluated in the OFT arena. After a 5-min familiarization phase with two identical objects and a 4-h interval, one object was replaced by a novel one. Exploration behavior (snout less than 2 cm from the object) was recorded for 5 min. The OFT arena and objects were cleaned with 75% ethanol between tests.

#### Elevated plus maze (EPM) test

Behavior was further evaluated using an EPM apparatus elevated 50 cm above the floor, consisting of two open and two enclosed arms connected by a central platform. Mice were placed in the center facing an open arm and recorded for 5 min. The number of entries into and time spent in the open arms were quantified. The maze was cleaned with 75% ethanol between tests.

#### Morris water maze (MWM) test

Spatial learning and memory were assessed in a circular pool (120 cm diameter, water at 22 ± 1 °C) surrounded by extra-maze visual cues. Mice underwent a 5-day hidden-platform training phase (four trials per day from different quadrants) to locate a platform submerged 1–2 cm below the surface. On day 6, a 60-s probe trial was conducted with the platform removed. Swimming trajectories, target platform crossings, and time spent in the target quadrant were recorded.

### Enzyme-linked immunosorbent assay (ELISA)

The Hippocampal tissues were homogenized in ice-cold RIPA supplemented with a protease inhibitor cocktail using a tissue homogenizer. Following homogenization, the lysates were centrifuged to remove cellular debris. The resulting cell-free supernatants were carefully collected. The concentrations of IL-1β, IL-6, TNF-*α*, and IL-22 in the supernatants were measured according to the manufacturers’ instructions (Elabscience, Wuhan, China). The optical density (OD) absorbance was recorded at 450 nm using a microplate reader (BioTek, Vermont, USA). The absolute concentrations of the cytokines were calculated based on the respective standard curves and expressed as pg/mL.

### Immunofluorescence staining

To evaluate hippocampal pro‑inflammatory microglial activation and postsynaptic damage, two independent TSA-based multiplex immunofluorescence assays were performed on separate 5 μm paraffin‑embedded coronal hippocampal sections from PW4 mice.

Mice were transcardially perfused with ice‑cold saline and 4% PFA. Brains were post‑fixed in 4% PFA for 24 h at 4 °C and embedded in paraffin. All sections underwent deparaffinization, heat‑induced antigen retrieval (citrate buffer, pH 6.0, 95 °C, 20 min), endogenous peroxidase quenching, and blocking with 5% BSA for 30 min at room temperature.

One set of sections was used for Iba‑1 and CD86 dual‑color co‑staining to detect pro‑inflammatory microglia. Sections were incubated with mixed primary antibodies (rabbit anti‑Iba‑1 and mouse anti‑CD86) overnight at 4 °C in a humidified chamber. After PBST washing, species‑matched Polymer‑HRP‑conjugated secondary antibodies were applied for signal amplification.

A second set of sections was processed for PSD‑95 staining to assess postsynaptic loss. Following the same pre‑treatment procedures, sections were incubated with anti‑PSD‑95 primary antibody overnight at 4 °C, followed by Polymer‑HRP‑conjugated secondary antibody‑mediated TSA signal amplification.

All sections were counterstained with DAPI. Confocal images were acquired in the hippocampal region (CA1, CA2, CA3, and DG) with consistent acquisition settings across groups. Using Fiji software, the percentage of Iba‑1⁺CD86⁺ double‑positive cells among total Iba‑1⁺ microglia was calculated to quantify pro‑inflammatory microglial activation. PSD‑95 mean fluorescence intensity (MFI) was measured to evaluate neuroinflammation‑related postsynaptic injury.

### Golgi staining

Dendritic spine density was quantified using standard Golgi-Cox impregnation. Briefly, fresh brains were immersed in Golgi-Cox solution (14 d, dark), cryoprotected (30% sucrose), and sectioned at 100–150 μm using a vibratome. Following standard development, dehydration, and clearing, sections were coverslipped. Well-isolated pyramidal neurons across hippocampal regions (CA1, CA2, CA3, and DG) were imaged using a light microscope (100× oil-immersion objective). Secondary or tertiary apical dendrites were analyzed via the ImageJ Analyze Skeleton plugin to calculate spines per 10 μm, quantitatively assessing synaptic loss.

### Transmission electron microscopy (TEM) analysis

To evaluate ultrastructural synaptic damage, mice were perfused with 2.5% glutaraldehyde/2% PFA. Hippocampal blocks (1 mm^3^) were post-fixed in 2.5% glutaraldehyde (overnight, 4 °C), osmicated (1% OsO_4_), dehydrated in graded acetone, and embedded in epoxy resin. Ultrathin sections (60–80 nm) were double-stained with uranyl acetate and lead citrate. Asymmetric excitatory synapses in the hippocampal neuropil were imaged via TEM. ImageJ software was used to quantify postsynaptic density (PSD) thickness.

### Western blot analysis

To quantify hippocampal PSD-95, AHR, CYP1A1, MAP2K3, and P-P38 expression, tissues from PW4 mice were homogenized in RIPA buffer containing protease and phosphatase inhibitors. Following centrifugation (12,000  rpm, 15  min, 4 °C), protein concentrations were determined via BCA assay. Proteins (30  μg) were resolved by 10% SDS-PAGE, transferred to PVDF membranes, and blocked (15 min). Membranes were incubated overnight (4 °C) with anti-PSD-95 anti-AHR, anti-CYP1A1, anti-MAP3K3, anti-P-P38 and anti-*β*-actin primary antibodies, followed by HRP-conjugated secondary antibodies (1 h, room temperature). Immunoreactive bands were visualized via ECL and quantified using ImageJ densitometry, normalized to *β*-actin.

### Targeted metabolomics and correlation analysis

Targeted metabolomics profiling (AQ700 panel) of frozen samples (50 μL) was performed using a QTRAP 6500+ LC–MS/MS system in MRM mode, following standard methanol-acetonitrile extraction and isotopic normalization. Absolute metabolite concentrations (nmol/L) were calculated via MultiQuant software. Following data filtering and log-transformation, multivariate analyses (PCA and OPLS-DA) were conducted using SIMCA (v18.0.1). Differential metabolites (VIP > 0, *P* < 0.05) were identified via *t*-tests or Mann-Whitney U tests.

### Gut metagenomics sequencing and microbiome analysis

Total genomic DNA was extracted from fecal samples using the Fecal Genome DNA Extraction Kit (AU46111-96, BioTek, China). DNA libraries were constructed with the TruSeq Nano DNA Library Preparation Kit-Set (#FC-121-4001, Illumina, USA) according to the manufacturer’s standard protocols. Metagenomic libraries were subsequently sequenced on the Illumina NovaSeq 6000 platform with a paired-end 150 bp (PE150) strategy at LC-Bio Technology Co., Ltd. (Hangzhou, China).

Raw sequencing reads were processed and quality-filtered using Fastp (v0.23.4) to remove adapter-contaminated sequences, low-quality bases, and ambiguous bases. Post-filtered high-quality reads were first aligned to the host reference genome using Bowtie2 (v2.2) to eliminate host-derived contaminating sequences. The remaining microbial clean reads were independently de novo assembled for each sample using MEGAHIT (v1.2.9) for subsequent taxonomic and functional analyses.

Coding sequence (CDS) prediction of assembled contigs was performed using MetaProdigal (v2.6.3). All predicted CDS sequences across samples were clustered and de-redundant via MMseqs2 (v15-6f452) to generate a comprehensive non-redundant unigene catalog. Taxonomic annotation of unigenes was implemented using DIAMOND (v0.9.14) against the NCBI NR database.

The Wilcoxon rank-sum test was applied to screen differentially abundant microbial taxa, with the significance threshold set at *P* < 0.05 and |log2-fold change| > 1. Functional annotation and profiling of microbial genes were performed based on the Kyoto Encyclopedia of Genes and Genomes (KEGG) database.

The 16S rRNA gene amplicon sequencing data are publicly available in the Sequence Read Archive (SRA) under BioProject accession No. PRJNA1460552. The shotgun metagenomic sequencing data analyzed in this study are also publicly available in SRA under BioProject accession No. PRJNA1510897.

### Fecal microbiota transplantation (FMT)

Fecal microbiota transplantation was conducted in a mouse model of necrotizing enterocolitis (NEC). Fecal inocula for FMT were harvested from juvenile donor mice at postnatal day 28 (P28). Donor mice had been subjected to NEC‑inducing insult and received β‑glucan treatment starting at postnatal day 12. No antibiotics, additional pharmacological agents, or other pretreatment were given to donors. Fecal microbiota suspensions were prepared by homogenizing 100  mg of fresh feces in 1 mL of sterile phosphate‑buffered saline (PBS) supplemented with 20% glycerol and 0.05% L‑cysteine, followed by vigorous vortexing. The mixture was centrifuged at 2000 rpm for 5 min at 4 °C, and the supernatant was harvested and stored at −80 °C until further use. Frozen fecal microbiota suspensions were thawed at room temperature immediately before transplantation. To reduce the load of endogenous intestinal bacteria, recipient mice were pretreated with a broad‑spectrum antibiotic cocktail (ampicillin 1 mg/mL, vancomycin 5 mg/mL, neomycin sulfate 10 mg/mL, metronidazole 10 mg/mL) 24 h prior to the first FMT. FMT was initiated on day 3 after the establishment of the NEC model; each recipient mouse received 200  μL of fecal microbiota suspension via oral gavage once daily for 14 consecutive days. Mice in the control group were administered an equal volume of sterile PBS via oral gavage at identical time points to ensure consistent experimental conditions across all groups.

### Hippocampal proteomic analysis

For investigating the effect of IAA intervention on the hippocampal protein expression profile in NEC mice, hippocampal tissues were collected from Control, NEC model, and NEC + IAA intervention groups (*n* = 5 per group, 15 independent biological samples total) for DIA-based quantitative proteomics analysis. Hippocampal tissues were homogenized in ice-cold SDT lysis buffer (4% SDS, 100 mM Tris-HCl, 1 mM DTT, pH 7.6) with protease and phosphatase inhibitors, boiled for 5 min, centrifuged at 14,000 × g for 15 min at 4 °C to collect supernatants, with total protein concentration quantified by BCA assay and integrity evaluated via SDS–PAGE. Equal amounts of protein (≈50 μg) from each sample were processed by FASP: reduced with 100  mM DTT at 37 °C for 1 h, alkylated with 50  mM iodoacetamide in the dark at room temperature for 30 min, digested with trypsin (enzyme-to-protein ratio 1:50, w/w) at 37 °C for 16 h, followed by peptide desalting with C18 SPE columns, lyophilization, and reconstitution in 0.1% formic acid. Peptides were separated by nano-LC coupled with a high-resolution mass spectrometer using a C18 column and a 90-min nonlinear gradient elution (mobile phase A: 0.1% FA in water; mobile phase B: 0.1% FA in 80% acetonitrile) at 300 nL/min, with MS data acquired in DIA mode (MS1 resolution: 60,000/120,000, mass range: 350–1500 m/z; HCD fragmentation, MS2 resolution: 30,000) and a DDA-generated QC sample library for reference. Raw MS data were analyzed via Spectronaut 15 against the Mus musculus UniProt database (FDR < 1%), with peptide quantification based on spectral peak AUC and data normalized by median normalization to calculate protein relative abundance. Bioinformatics analyses were performed using R software (v4.0.0): PCA for sample clustering, two-tailed Student's t-test to identify DEPs (FC > 1.2 or < 0.83, *P* < 0.05), hierarchical clustering/heatmaps for DEP visualization, and GO/KEGG enrichment analysis to explore regulated signaling pathways.

### Drug administration

Following NEC induction, surviving mice were randomly assigned to groups: Control, NEC, NEC + L-βglucan, NEC + H-βglucan, NEC + IAA, and NEC + IAA + CH223191. The NEC + L-βglucan and NEC + H-βglucan groups received daily intragastric βglucan (Angel Yeast Co., Ltd. (China); 10 mg/kg or 100 mg/kg; formulated in PBS). The NEC + IAA group received daily intragastric IAA (20 mg/kg; formulated in 1% DMSO, 10% PEG300, 5% Tween-80, and 84% saline) for 2 weeks. To block AHR signaling, the NEC + IAA + CH223191 group was intraperitoneally injected with the AHR antagonist CH223191 (10  mg/kg; vehicle: 10% DMSO, 40% PEG300, 5% Tween-80, 45% saline) 1 h prior to each IAA administration. Control and NEC mice received equivalent volumes of corresponding vehicles via identical routes.

### Statistical analysis

Statistical analyses and data visualization were performed using GraphPad Prism (v10.5). Normality of data distribution was evaluated via the Shapiro-Wilk test. Normally distributed variables are presented as mean  ±  standard deviation (SD) or standard error of the mean (SEM), whereas non-normally distributed data are expressed as medians with interquartile ranges. Two-group comparisons were conducted using an unpaired, two-tailed Student’s *t*-test or a Mann-Whitney U test, as appropriate. For multiple-group comparisons, a one-way analysis of variance (ANOVA) followed by Tukey’s post-hoc test was utilized. For datasets that deviated from normal distribution or contained prominent outliers, we switched to non‑parametric analyses, including Kruskal–Wallis test followed by Dunn’s multiple‑comparison post‑hoc test. Correlations were assessed using Pearson or Spearman rank correlation coefficients.

## Results

### *β*-glucan ameliorates cognitive impairment in NEC mice

To explore the neuroprotective effect of *β*-glucan on NEC-induced behavioral deficits, we systematically evaluated cognitive function using a battery of behavioral tests.

In the elevated plus‑maze (EPM) test, NEC mice exhibited significantly reduced open‑arm exploratory behavior. Both low‑dose and high‑dose β‑glucan interventions significantly restored the moving distance (*P* < 0.01, *P* < 0.01), number of open‑arm entries (*P* < 0.01, *P* < 0.05), and residence time (*P* < 0.05, *P* < 0.05) of mice in the open‑arm zone (Figure 1B,C). Notably, no significant statistical difference was observed between the low-dose and high-dose *β*-glucan groups. In the open‑field test (OFT), compared with the normal group, NEC mice exhibited reduced exploratory behavior in the central area (*P* < 0.0001) and decreased total spontaneous activity (*P* < 0.001). β‑glucan intervention could effectively ameliorate the above behavioral abnormalities, and the therapeutic effects were comparable between the two dose groups (Figure 1D,E).

**Figure 1.**
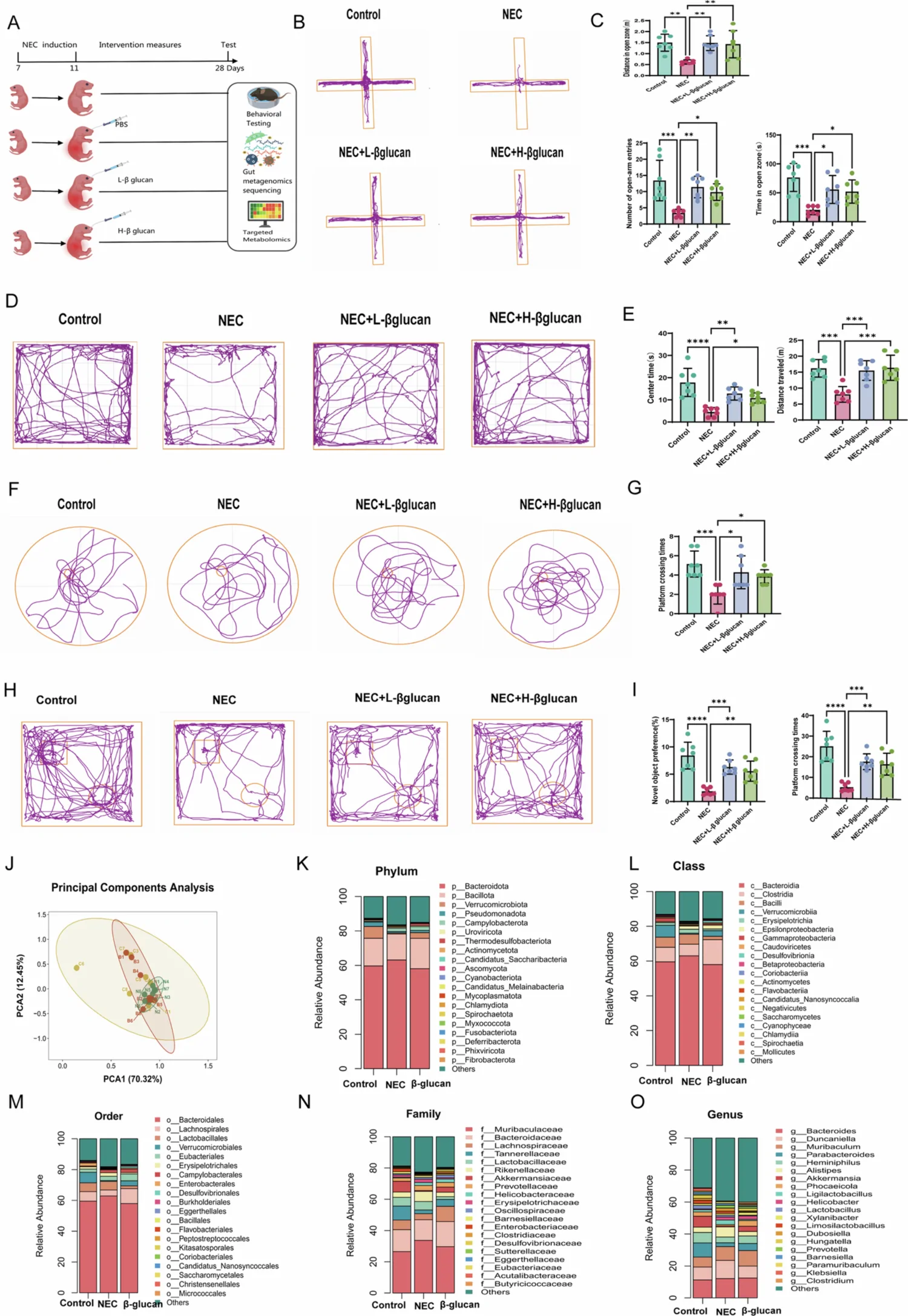
(A) Experimental design schematic. (B, C) Representative movement trajectories (B) and quantitative analysis (C) of the elevated plus maze (EPM) test. *N* = 7 per group. (D, E) Representative movement trajectories (D) and quantitative analysis (E) of the open field test (OFT). *N* = 7 per group. (F, G) Representative search trajectories (F) and quantitative analysis (G) of the Morris water maze (MWM) test. *N* = 7 per group. (H, I) Representative exploration trajectories (H) and quantitative analysis (I) of the novel object recognition (NOR) test. *N* = 7 per group. (J) Principal components analysis. The relative abundance of significantly altered bacterial taxa at the phylum (K), class (L), order (M), family (N), and genus (O) levels. Groups: Control, NEC, NEC + L-βglucan (low-dose), and NEC + H-βglucan (high-dose). Data are presented as mean ± SD. *P < 0.05, **P < 0.01, ***P < 0.001, ****P < 0.0001, determined by one-way ANOVA followed by Tukey^’^s post-hoc test.

Morris water maze (MWM) analysis showed that compared with the normal group, mice with necrotizing enterocolitis (NEC) exhibited impaired spatial memory, manifested as disordered search trajectories and fewer platform‑crossing times (*P* < 0.001). Compared with the NEC group, the β‑glucan group had significantly increased platform‑crossing times (*P* < 0.05, *P* < 0.05), and there was no statistically significant difference between the low‑dose β‑glucan group and the high‑dose β‑glucan group (Figure 1F,G). In the Novel Object Recognition (NOR) test, compared with the normal group, mice with necrotizing enterocolitis (NEC) showed impaired recognition memory, decreased preference for novel objects (*P* < 0.0001), and shortened exploration time (*P* < 0.0001). Treatment with β‑glucan markedly alleviated cognitive dysfunction, and the therapeutic effects of low‑dose and high‑dose β‑glucan were comparable (Figure 1H,I).

These results demonstrate that *β*-glucan effectively ameliorates cognitive impairment in NEC mice, with no significant statistical difference between low-dose and high-dose interventions.

### *β*-glucan remodels gut microbiota composition in NEC mice

Principal‑component analysis (PCA) was performed to compare gut microbial community structure across groups (Figure 1J). PCA1 and PCA2 accounted for 70.32% and 12.45% of total variance. The control group was clearly separated from the NEC group, indicating marked gut microbiota disturbance after NEC injury. The β‑glucan group partially shifted toward the control cluster, suggesting partial restoration of the perturbed microbial community by β‑glucan intervention. Partial overlap between *β*-glucan and NEC was still observed, showing that full recovery of microbiota composition was not achieved.

At phylum and class levels (Figure 1K,L), the Control group maintained a balanced microbial community predominantly composed of *Bacteroidota* (*Bacteroidia*) and *Bacillota* (*Clostridia, Bacilli*). Following NEC injury, we observed marked expansion of inflammation‑associated taxa including (*Gammaproteobacteria*, *Epsilonproteobacteria*), *Campylobacterota*, and *Thermodesulfobacteriota* (*Desulfovibrionia*). Relative to the NEC group, β‑glucan intervention restored community structure, with elevated *Verrucomicrobiota* (*Verrucomicrobia*) and reduced abundance of pathogenic phyla and classes, shifting the overall profile toward that seen in Control animals.

Consistent changes were observed across order, family, and genus ranks (Figure 1M–O). The Control group was enriched in beneficial commensal taxa, such as *Bacteroidales*, *Lactobacillales*, *Muribaculaceae*, *Akkermansiaceae*, *Lactobacillaceae*, *Akkermansia*, *Muribaculum*, *Alistipes*, and *Duncaniella*. NEC triggered abnormal enrichment of pro‑inflammatory taxa, including *Campylobacterales*, *Enterobacterales*, *Helicobacteraceae*, *Enterobacteriaceae*, *Desulfovibrionaceae*, *Helicobacter*, and *Klebsiella*. These NEC‑driven shifts were significantly mitigated by β‑glucan, which recovered the abundance of beneficial commensal taxa and suppressed pro‑inflammatory genera. At the species level, beneficial commensal taxa including *Akkermansia muciniphila*, *Lactobacillus johnsonii*, *Ligilactobacillus murinus*, and *Limosilactobacillus* reuteri were abundant in Control mice, nearly depleted under NEC conditions, and significantly restored after β‑glucan intervention.

Linear discriminant analysis effect size (LEfSe) (Figure 2A) further identified discriminative taxa between the NEC and β‑glucan groups. Taxa enriched in the β‑glucan group covered the full lineage of *Verrucomicrobiota*‑*Verrucomicrobiia*‑*Verrucomicrobiales*‑*Akkermansiaceae*‑*Akkermansia*‑*Akkermansia muciniphila*, alongside *Lactobacillales*, *Lactobacillaceae*, *Alistipes*, *Duncaniella*, and *Muribaculaceae*. By contrast, the NEC group was characterized by enrichment of pro‑inflammatory taxa including *Clostridia*, *Lachnospirales*, *Lachnospiraceae*, *Desulfovibrionia*, *Desulfovibrionales*, *Desulfovibrionaceae*, *Taurinivorans*, and *Phocaeicola sartorii*.

**Figure 2.**
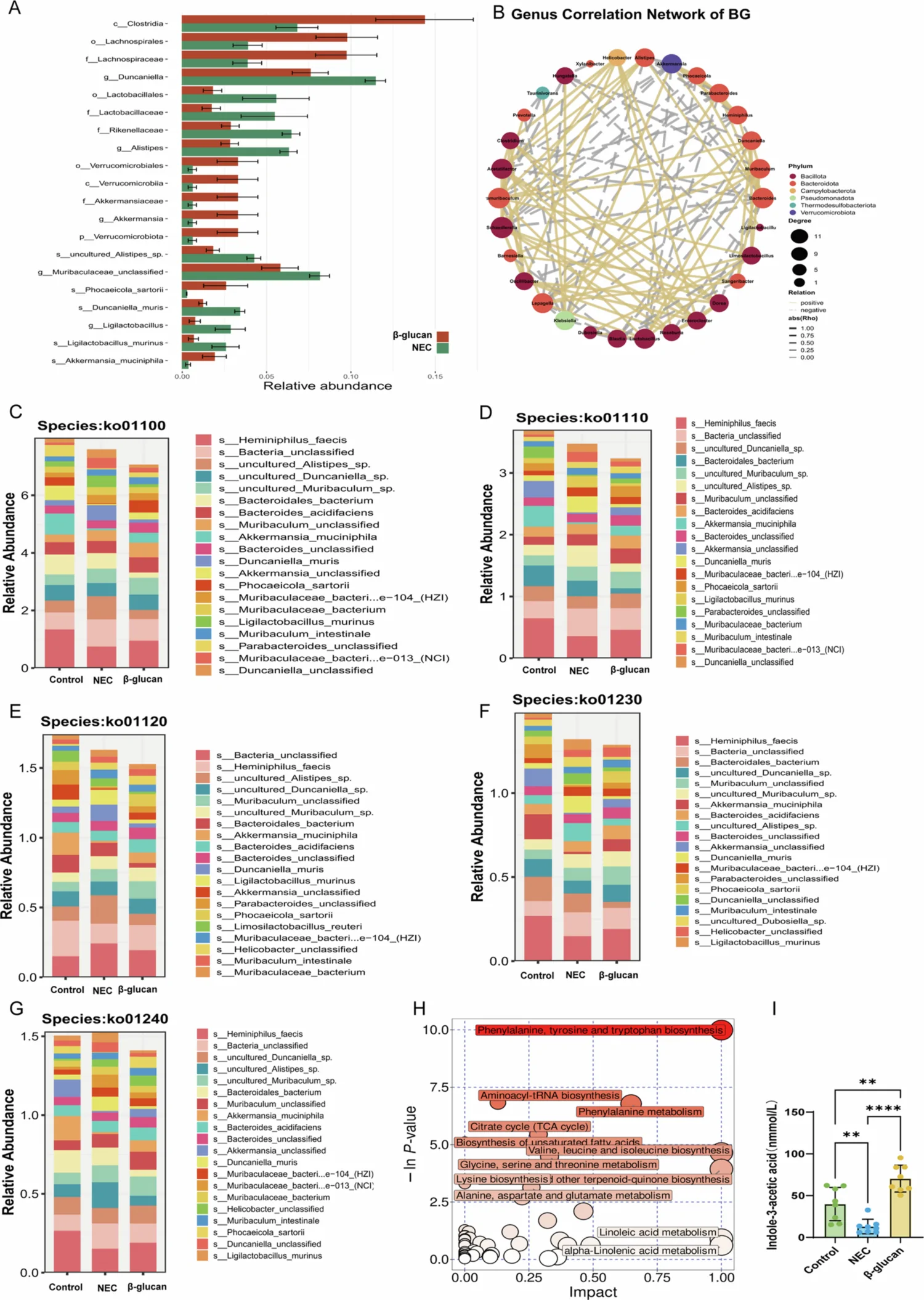
(A) LEfSe differential flora analysis between the *β*-glucan and NEC groups. (B) The level of *β*-glucan group microbiota correlation network. (C–E) Pathways related to sugar metabolism. (F) Amino acid metabolic pathway. (G) Arginine/Proline metabolism. (H) Bubble plot of metabolic pathway enrichment analysis. (I) Quantification of indole-3-acetic acid in the control, NEC, and *β*-glucan groups. *N* = 8 per group. Data are presented as mean ± SD. Statistical significance was determined by one-way ANOVA followed by Tukey’s post-hoc test.

We next constructed a genus‑level correlation‑network (|ρ| ≥ 0.8) for the β‑glucan‑treated group (Figure 2B). This network encompassed major phyla (*Bacteroidota*, *Bacillota*, *Verrucomicrobiota*, and *Pseudomonadota*), with *Muribaculum* (degree = 11) and *Bacteroides* (degree = 9) as the two primary hub genera, consistent with core commensal taxa in the Control group. Positive correlations dominated the network, indicating a synergistic, stable microbial interaction pattern reminiscent of the physiological state. Key beneficial commensal genera such as *Akkermansia*, *Lactobacillus*, and *Alistipes* were integrated within this network.

We then assessed species‑level contributions to five core metabolic pathways (ko01100, ko01110, ko01120, ko01230, and ko01240) (Figure 2C–G). Major contributing species included *Akkermansia muciniphila*, *Muribaculaceae bacterium*, *Heminiphilus faecis*, Duncaniella muris, and *Ligilactobacillus murinus*. The relative abundance of these pathway‑contributing species was highest in the Control group, lowest in the NEC group, and significantly restored in the β‑glucan group, suggesting an association between β‑glucan treatment, recovery of beneficial commensal taxa, and recovery of predicted microbial metabolic capacity.

Collectively, these data show that β‑glucan largely counteracts NEC‑triggered gut dysbiosis. It partially restores microbial community composition, inter‑bacterial interaction patterns, and predicted metabolic profiles toward the state observed in Control mice, pointing to gut microbiota as a potential mediator of β‑glucan‑conferred protection against long‑term cognitive impairment following NEC.

### *β*-glucan modulates tryptophan metabolism in NEC mice, with indole-3-acetic acid (IAA) as the key regulator

Metabolic pathway enrichment analysis showed that the phenylalanine, tyrosine, and tryptophan biosynthesis pathway was the most significantly altered in NEC mice treated with *β*-glucan (Figure 2H), exhibiting the highest impact value and statistical significance (as indicated by −ln *P*-value). Subsequent quantification of key tryptophan metabolites demonstrated that, compared with the Control group, the levels of indole-3-acetic acid (IAA) were significantly decreased in the NEC group (Figure 2I; *P* < 0.01). *β*-Glucan intervention significantly restored the concentrations of IAA (Figure 2I; *P* < 0.0001), reaching levels significantly higher than those in the NEC group. Other indole derivatives, including indole-3-carboxaldehyde and indole-3-carboxylic acid, exhibited a similar trend of depletion in the NEC group and restoration following *β*-glucan treatment, albeit with a less pronounced effect than that of IAA (Supplementary Figure). These data indicate that *β*-glucan comprehensively reverses NEC-induced dysregulation of the tryptophan metabolic pathway, with IAA emerging as the most critical and responsive metabolite.

### Fecal microbiota transplantation (FMT) confirms the gut microbiota-mediated neuroprotective effects of *β*-glucan

To verify whether the gut microbiota mediates the *β*-glucan-induced cognitive improvement, we performed fecal microbiota transplantation (FMT) of gut microbiota from *β*-glucan-treated mice into NEC mice. In the EPM test, mice in the NEC+FMT group exhibited a significant increase in open-arm traveling distance (Figure 3B,C; *P* < 0.01), frequency of open-arm entries (Figure 3B,C; *P* < 0.05), and time spent in the open arms (Figure 3B,C; *P* < 0.01) compared with the NEC group – indicating attenuated anxiety-like behaviors. In the OFT, NEC + FMT mice showed a significant increase in time spent in the central zone (Figure 3D,E; *P* < 0.05) and total locomotor distance (Figure 3D,E; *P* < 0.01). In the MWM test, the time spent in the target quadrant was significantly prolonged in the NEC + FMT group (Figure 3H,I; *P* < 0.01). Furthermore, the NOR test revealed that NEC + FMT mice exhibited a higher novel object preference index (Figure 3F,G; *P* < 0.05) and longer novel object exploration time (Figure 3F,G; *P* < 0.05). These results demonstrate that transplantation of gut microbiota from *β*-glucan-treated mice is sufficient to alleviate cognitive impairment in NEC mice, confirming a causal role of the gut microbiota in mediating the neuroprotective effects of *β*-glucan.

**Figure 3.**
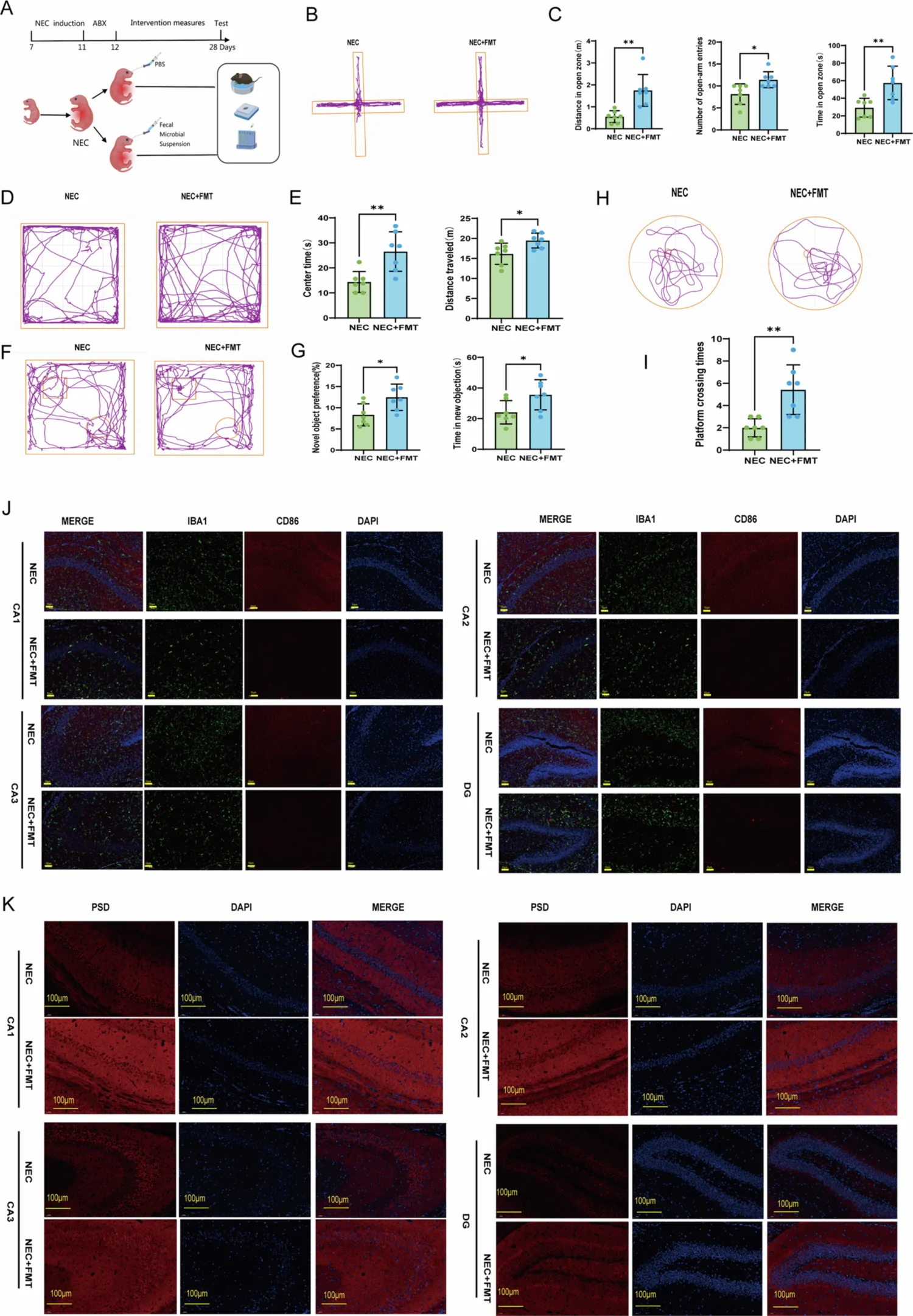
(A) Experimental design schematic. (B, C) Representative movement trajectories (B) and quantitative analysis (C) of the elevated plus maze (EPM) test. *N* = 7 per group. (D, E) Representative movement trajectories (D) and quantitative analysis (E) of the open field test (OFT). *N* = 7 per group. (F, G) Representative exploration trajectories (F) and quantitative analysis (G) of the novel object recognition (NOR) test. *N* = 7 per group. (H, I) Representative search trajectories (H) and quantitative analysis (I) of the Morris water maze (MWM) test. *N* = 7 per group. (J) Representative immunofluorescence images of IBA1 (green, microglia marker), CD86 (red), and DAPI (blue, nuclei) staining in the CA1, CA2, CA3, and DG regions of the hippocampus in NEC and NEC + FMT mice. Scale bar = 50 μm. (K) Representative immunofluorescence images of PSD95 (red, postsynaptic marker) and DAPI (blue, nuclei) staining in the CA1, CA2, CA3, and DG regions of the hippocampus in NEC and NEC + FMT mice. Scale bar = 100 μm. Data are presented as mean ± SD. Statistical significance was determined by unpaired Student’s t-test. *P < 0.05, **P < 0.01; ***P < 0.001.

#### FMT suppresses hippocampal microglial activation in NEC mice

To investigate the neuroinflammatory mechanism underlying FMT-induced cognitive improvement, we assessed microglial activation in the hippocampus via IBA1 and CD86 immunofluorescence staining (Figure 3J). Compared with the NEC group, the NEC + FMT group exhibited a marked reduction in IBA1- and CD86-positive signals across all hippocampal subregions (CA1, CA2, CA3, and DG). Quantitative analysis further confirmed that the percent of IBA1^+^CD86^+^ microglia was significantly lower in the NEC + FMT group than in the NEC group across all subregions (Figure 4A; ****P < 0.0001 for all comparisons). These results indicate that gut microbiota intervention via FMT effectively inhibits excessive microglial activation, thereby mitigating hippocampal neuroinflammation in NEC mice.

**Figure 4.**
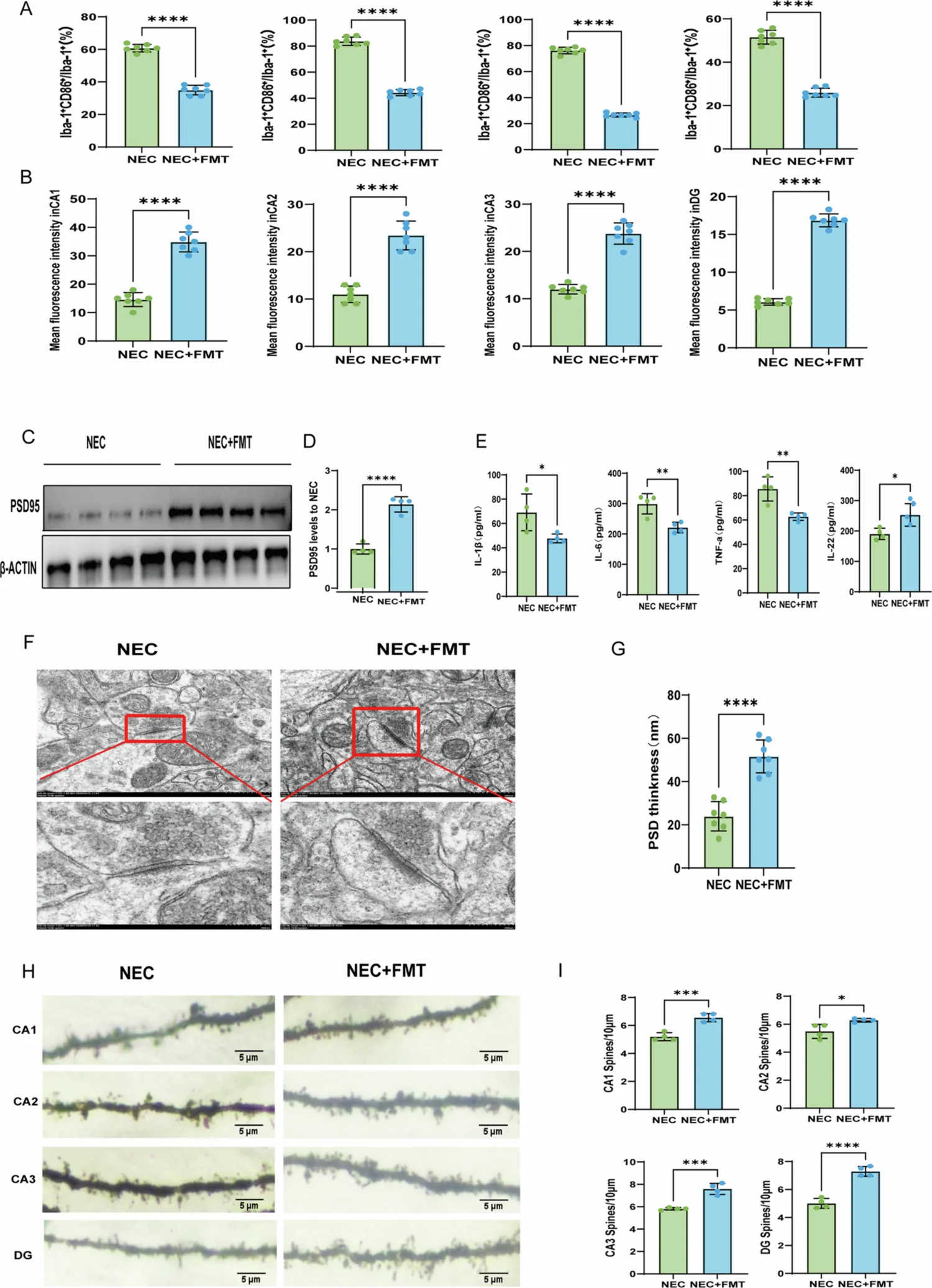
(A) Analysis of the percentage of IBA1^+^CD86^+^ cells in four regions of the hippocampus. *N* = 7 per group. (B) Quantitative analysis of PSD95 mean fluorescence intensity in the four hippocampal subregions. *N* = 7 per group. (C, D) Western blot analysis of PSD95 protein expression in the hippocampus. *β*-actin was used as a loading control. Quantification of PSD95 levels normalized to *β*-actin is shown in (D). *N* = 4 per group. (E) ELISA analysis of pro-inflammatory cytokines (IL-1β, IL-6, and TNF-*α*) and the anti-inflammatory cytokine (IL-22) levels in the hippocampus of NEC and NEC + FMT mice. *N* = 4 per group. (F, G) Transmission electron microscopy (TEM) images (F) and quantitative analysis (G) of postsynaptic density (PSD) thickness in the hippocampus. Scale bar = 500 nm (top) and 200 nm (bottom). *N* = 7 per group. (H, I) Representative Golgi staining images (H) and quantitative analysis (I) of dendritic spine density in the hippocampal CA1, CA2, CA3, and DG regions. Scale bar = 5 μm. *N* = 4 per group. Data are presented as mean ± SD. ****P < 0.0001, determined by unpaired Student’s t-test.

#### FMT restores hippocampal synaptic function in NEC mice via upregulating PSD95 expression

To assess the effect of FMT on synaptic integrity in NEC mice, we evaluated the expression of postsynaptic density protein 95 (PSD95) – a key marker of synaptic function – via immunofluorescence staining and Western blot analysis. Immunofluorescence images showed that PSD95 (red fluorescence) signals were markedly reduced in the hippocampal CA1, CA2, CA3, and DG regions of the NEC group, whereas they were significantly enhanced in the NEC+FMT group (Figure 3K). Quantitative analysis further confirmed that the mean fluorescence intensity of PSD95 in all four hippocampal subregions was significantly higher in the NEC+FMT group than in the NEC group (Figure 4B; *P* < 0.0001 for all comparisons). Western blot analysis (Figure 4C,D) consistently confirmed that PSD95 protein expression was dramatically upregulated in the NEC+FMT group compared with the NEC group (*P* < 0.0001). These results indicate that FMT effectively restores synaptic structure and function in the hippocampus of NEC mice, which may contribute to the observed improvement in cognitive function.

#### FMT attenuates neuroinflammation and restores synaptic plasticity in NEC mice

To explore the mechanisms underlying FMT-induced synaptic recovery, we first quantified the levels of inflammatory cytokines in the hippocampus. Compared with the NEC group, the concentrations of the pro-inflammatory cytokines IL-1β (*P* < 0.05), IL-6 (*P* < 0.01), and TNF-α (*P* < 0.01) were significantly reduced in the NEC+FMT group, while the anti-inflammatory cytokine IL-22 (*P* < 0.05) was markedly upregulated (Figure 4E) – demonstrating that FMT effectively mitigated hippocampal neuroinflammation in NEC mice.

Transmission electron microscopy (TEM) was used to assess synaptic ultrastructure. The postsynaptic density (PSD) was notably thinner and less distinct in the NEC group, whereas it was significantly thickened and structurally intact in the NEC+FMT group (Figure 4F). Quantitative analysis confirmed that PSD thickness was dramatically increased in the NEC+FMT group (Figure 4G; *P* < 0.0001).

Furthermore, Golgi staining was performed to evaluate dendritic spine density in the hippocampal CA1, CA2, CA3, and DG regions. NEC mice displayed sparse and underdeveloped dendritic spines, while FMT treatment significantly increased spine density across all four subregions (Figure 4H,I; *P* < 0.05 to *P* < 0.001). These findings demonstrate that FMT suppresses neuroinflammation, restores synaptic ultrastructure, and enhances synaptic plasticity in the hippocampus of NEC mice.

#### IAA ameliorates cognitive impairment in NEC mice via the aryl hydrocarbon receptor (AHR) signaling pathway

To validate the neuroprotective potential of indole-3-acetic acid (IAA) and clarify whether these effects are mediated by the aryl hydrocarbon receptor (AHR) signaling pathway, we established four experimental groups: Control, necrotizing enterocolitis (NEC), NEC + IAA, and NEC+IAA+CH223191 (a selective AHR inhibitor). Behavioral assessments were first performed to evaluate cognitive function. In the elevated plus maze (EPM) test, NEC mice exhibited significant reductions in open-arm travel distance (*P* < 0.01), open-arm entry frequency (*P* < 0.01), and open-arm residence time (*P* < 0.05) compared with Control mice. These behavioral deficits were markedly ameliorated by IAA administration (*P* < 0.05 vs. NEC group), whereas concurrent treatment with CH223191 completely abrogated the therapeutic effects of IAA (*P* < 0.05 to 0.01 vs. NEC+IAA group) (Figure 5B,C). Consistent findings were observed in the open field test (OFT): NEC mice showed decreased central zone residence time and total locomotor activity (both *P* < 0.01), which were effectively restored by IAA treatment – an improvement that was blocked by AHR inhibition (Figure 5D,E).

**Figure 5.**
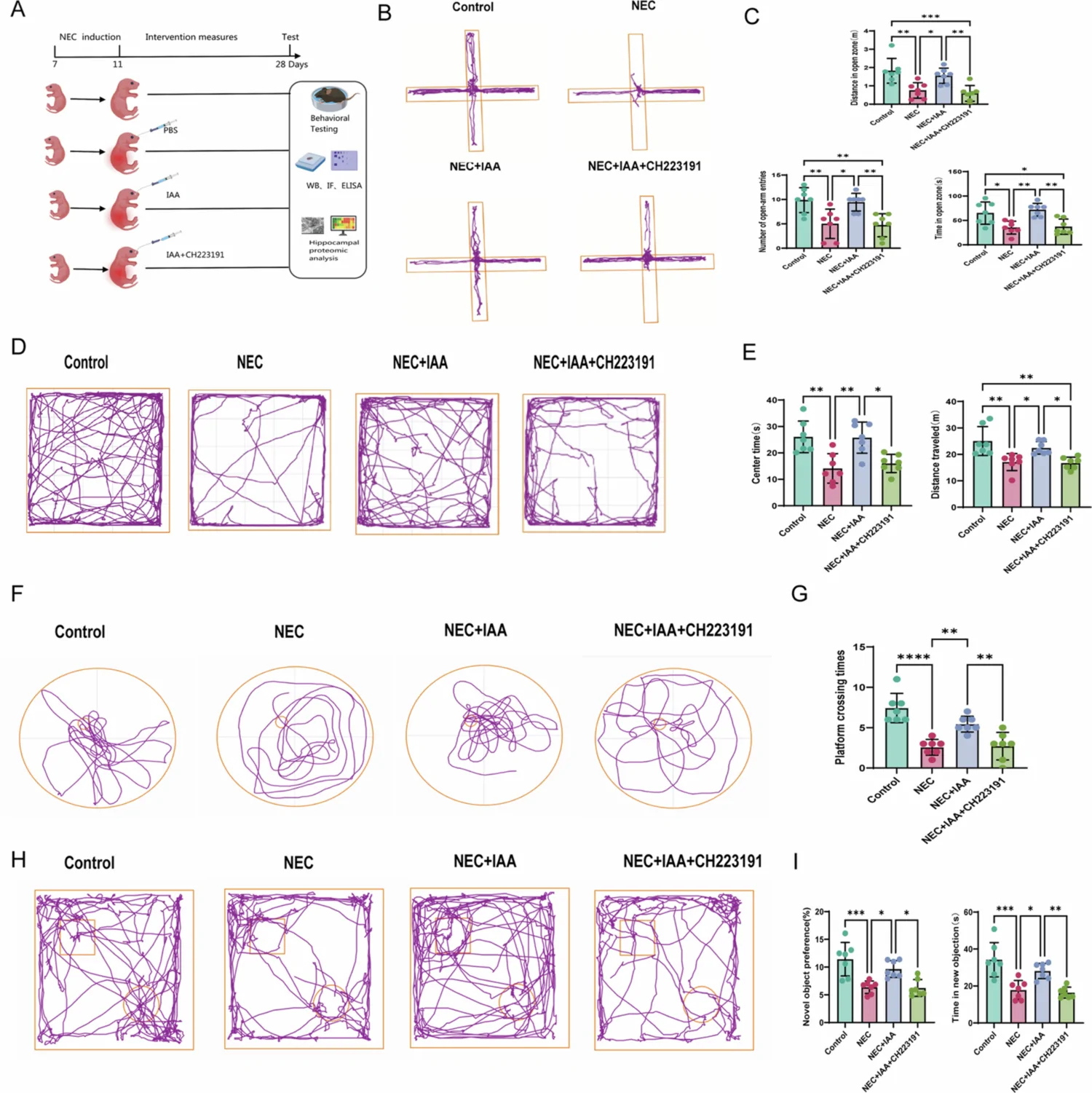
(A) Experimental design schematic. (B, C) Representative movement trajectories (B) and quantitative analysis (C) of the elevated plus maze (EPM) test. *N* = 7 per group. (D, E) Representative movement trajectories (D) and quantitative analysis (E) of the open field test (OFT). *N* = 7 per group. (F, G) Representative search trajectories (F) and quantitative analysis (G) of the Morris water maze (MWM) test. *N* = 7 per group. (H, I) Representative exploration trajectories (H) and quantitative analysis (I) of the novel object recognition (NOR) test. *N* = 7 per group. Groups: Control, NEC, NEC + IAA, and NEC + IAA + CH223191. Data are presented as mean ± SD. *P < 0.05, **P < 0.01, ***P < 0.001, ****P < 0.0001, determined by one-way ANOVA followed by Tukey’s post-hoc test.

Subsequent cognitive function evaluations further confirmed the AHR-dependent neuroprotective role of IAA. In the Morris water maze (MWM) test, NEC mice displayed fewer platform crossings (*P* <  0.0001 vs. Control), indicating impaired spatial learning and memory. IAA intervention significantly increased this index (*P* < 0.01 vs. NEC), while co-treatment with CH223191 reversed the beneficial effect (Figure 5F,G). In the novel object recognition (NOR) test, NEC mice exhibited reduced novel object preference and exploration time (both *P* < 0.001 vs. Control), and these cognitive dysfunctions were notably alleviated by IAA (*P* < 0.05 vs. NEC). Pretreatment with the AHR inhibitor abolished these protective outcomes (Figure 5H,I). Collectively, these behavioral data demonstrate that IAA exerts cognitive-protective effects against NEC-induced brain damage in an AHR-dependent manner.

To explore the underlying cellular mechanism, hippocampal microglial activation was assessed via immunofluorescence staining for ionized calcium-binding adapter molecule 1 (IBA1) and CD86, a well-established marker of microglial activation. Representative images revealed a marked increase in the percent of IBA1^+^ CD86^+^ microglia in the hippocampal CA1, CA2, CA3, and dentate gyrus (DG) regions of NEC mice, indicative of aberrant microglial activation. IAA treatment significantly reduced the percent of IBA1^+^ CD86^+^ microglia, an effect that was fully abrogated by CH223191 intervention (Figure 6A). Quantitative analysis confirmed that the percent of IBA1+ CD 86+ microglia was significantly higher in the NEC group than in the Control group across all hippocampal subregions (Figure 6B, *P* < 0.0001). IAA supplementation notably reduced the percent of IBA1+ CD86+ microglia (*P* < 0.05–0.0001 vs. NEC), and this reduction was completely reversed by AHR inhibition (Figure 6B; *P* < 0.05 to 0.0001 vs. NEC+IAA). These results indicate that IAA suppresses excessive hippocampal microglial activation through the AHR signaling pathway.

**Figure 6.**
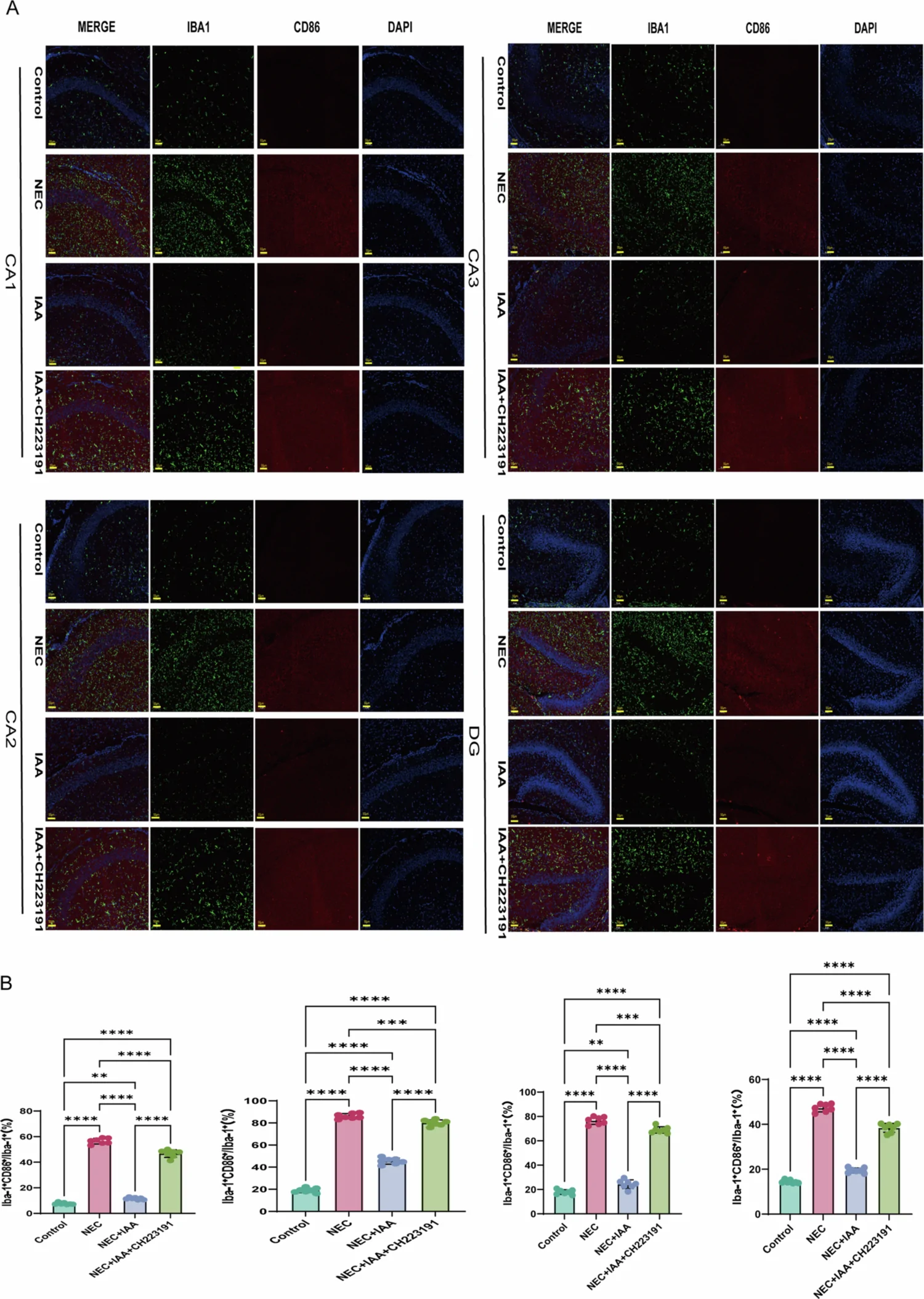
(A) Representative immunofluorescence images of IBA1 (green, microglia marker) CD86(red)and DAPI (blue, nuclei) staining in the CA1, CA2, CA3, and DG regions of the hippocampus in NEC and NEC + FMT mice. Scale bar = 50 μm. *N* = 7 per group. (B) Analysis of the percentage of IBA1^+^CD86^+^ cells in four regions of the hippocampus. *N* = 7. Groups: Control, NEC, NEC + IAA, and NEC + IAA + CH223191. *N* = 7 per group. Data are presented as mean ± SD. *P < 0.05, **P < 0.01, ***P < 0.001, ****P < 0.0001, determined by one-way ANOVA followed by Tukey’s post-hoc test.

Given the critical role of synaptic integrity in maintaining cognitive function, we next evaluated the expression of postsynaptic density protein 95 (PSD95), a key marker of synaptic structural stability. Immunofluorescence staining showed that PSD95 fluorescence intensity was prominently diminished in multiple hippocampal regions of NEC mice, reflecting severe synaptic structural damage. IAA treatment effectively upregulated hippocampal PSD95 expression and enhanced its fluorescent signals, whereas AHR activity blockade reversed the synaptic protective effect of IAA (Figure 7A). Quantitative analysis further confirmed that the mean fluorescence intensity of PSD95 was significantly lower in the NEC group than in the Control group (*P* < 0.0001). IAA intervention markedly restored PSD95 expression (*P* < 0.0001 vs. NEC), and this restorative effect was completely abolished by CH223191 (Figure 7B; *P* < 0.01–0.0001 vs. NEC+IAA).

**Figure 7.**
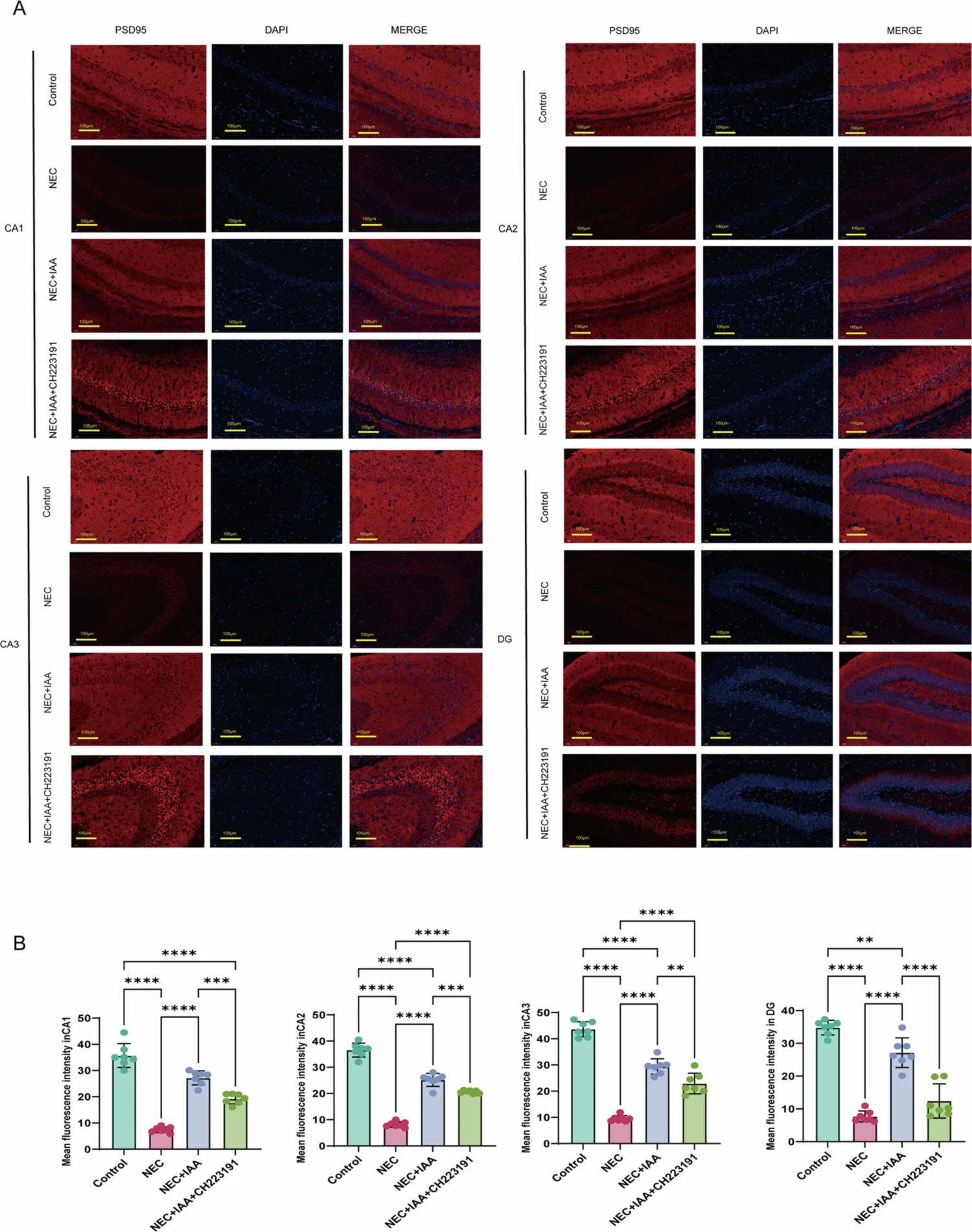
(A) Representative immunofluorescence images of PSD95 (red, postsynaptic marker) and DAPI (blue, nuclei) staining in the CA1, CA2, CA3, and DG regions of the hippocampus in Control, NEC, NEC + IAA, and NEC + IAA + CH223191 mice. Scale bar = 100 μm. *N* = 7 per group. (B) Quantitative analysis of PSD95 mean fluorescence intensity in the four hippocampal subregions. *N* = 7 per group. Groups: Control, NEC, NEC + IAA, and NEC + IAA + CH223191. Data are presented as mean ± SD. *P < 0.05, **P < 0.01, ***P < 0.001, ****P < 0.0001, determined by one-way ANOVA followed by Tukey’s post-hoc test.

We further systematically investigated the regulatory effects of IAA on hippocampal neuroinflammation and synaptic plasticity via the AHR pathway. Quantification of hippocampal inflammatory cytokines revealed that the levels of pro-inflammatory factors (IL-1β, IL-6, and TNF-*α*) were significantly elevated in NEC mice (Figure 8A, *P* <0.01–0.0001), accompanied by a marked reduction in the anti-inflammatory cytokine IL-22. IAA treatment effectively inhibited the secretion of pro-inflammatory cytokines and upregulated IL-22 expression (*P* <  0.05–0.0001 vs. NEC), and these anti-inflammatory properties were abrogated following AHR inhibition (Figure 8A). Transmission electron microscopy (TEM) observations showed that the postsynaptic density (PSD) was notably thinner in NEC mice, and IAA treatment significantly thickened the PSD structure – an improvement that was reversed by CH223191 (Figure 8B,C). Additionally, Golgi staining demonstrated that dendritic spine density was significantly decreased in all hippocampal subregions of NEC mice (*P* < 0.0001 vs. Control). IAA effectively increased dendritic spine density (*P* < 0.05–0.0001 vs. NEC), while AHR inhibition eliminated this positive regulatory effect (Figure 8D,E). Taken together, these findings demonstrate that IAA alleviates hippocampal neuroinflammation and restores impaired synaptic plasticity in NEC mice through an AHR-dependent mechanism.

**Figure 8.**
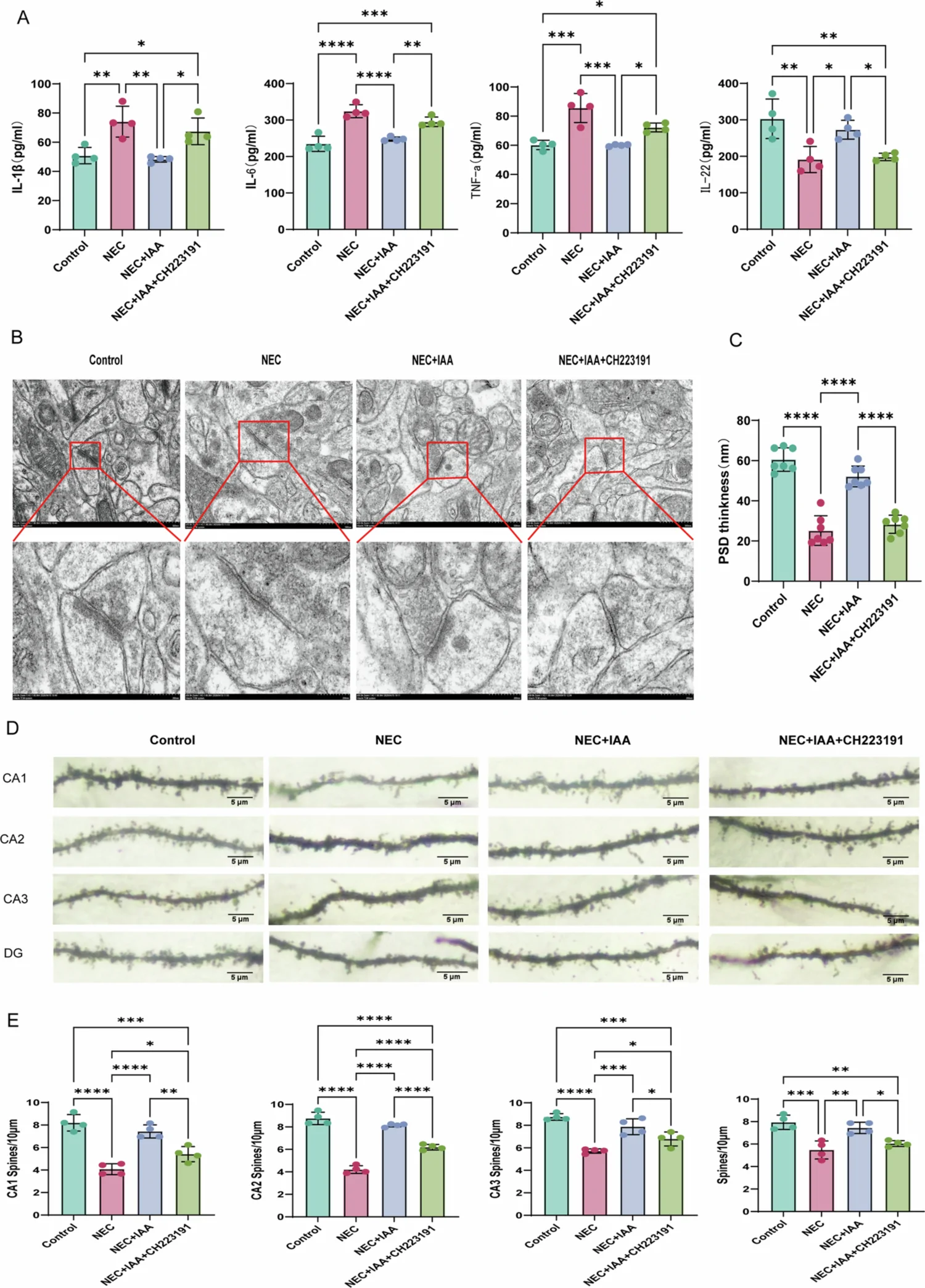
(A) ELISA analysis of pro-inflammatory cytokines (IL-1β, IL-6, and TNF-*α*) and anti-inflammatory cytokine (IL-22) levels in the hippocampus. *N* = 4 per group. (B, C) Transmission electron microscopy (TEM) images (B) and quantitative analysis (C) of postsynaptic density (PSD) thickness in the hippocampus. Scale bar = 500 nm (top) and 200 nm (bottom). *N* = 7 per group. (D, E) Representative Golgi staining images (D) and quantitative analysis (E) of dendritic spine density in the hippocampal CA1, CA2, CA3, and DG regions. Scale bar = 5 μm. *N* = 4 per group. Groups: Control, NEC, NEC + IAA, and NEC + IAA + CH223191. Data are presented as mean ± SD. *P < 0.05, **P < 0.01, ***P < 0.001, ****P < 0.0001, determined by one-way ANOVA followed by Tukey’s post-hoc test.

#### Hippocampal proteomics reveals that IAA improves synaptic function via the AHR/MAP2K3/p38 signaling pathway

To investigate the molecular mechanism underlying the neuroprotective effect of IAA, we conducted hippocampal proteomic analysis followed by pathway validation.

Gene ontology (GO) enrichment analysis demonstrated that differentially expressed proteins were primarily enriched in biological processes associated with neural development, including glial cell development, pallium development, chromatin remodeling, forebrain development, and central nervous system development (Figure 9A). Kyoto Encyclopedia of Genes and Genomes (KEGG) pathway analysis revealed significant enrichment of the MAPK signaling pathway and the PI3K-Akt signaling pathway (Figure 9B). A chord diagram further visualized the key hub proteins, with MAP2K3 (the upstream kinase of p38) identified as a central node connecting synaptic and signaling pathways (Figure 9C).

**Figure 9.**
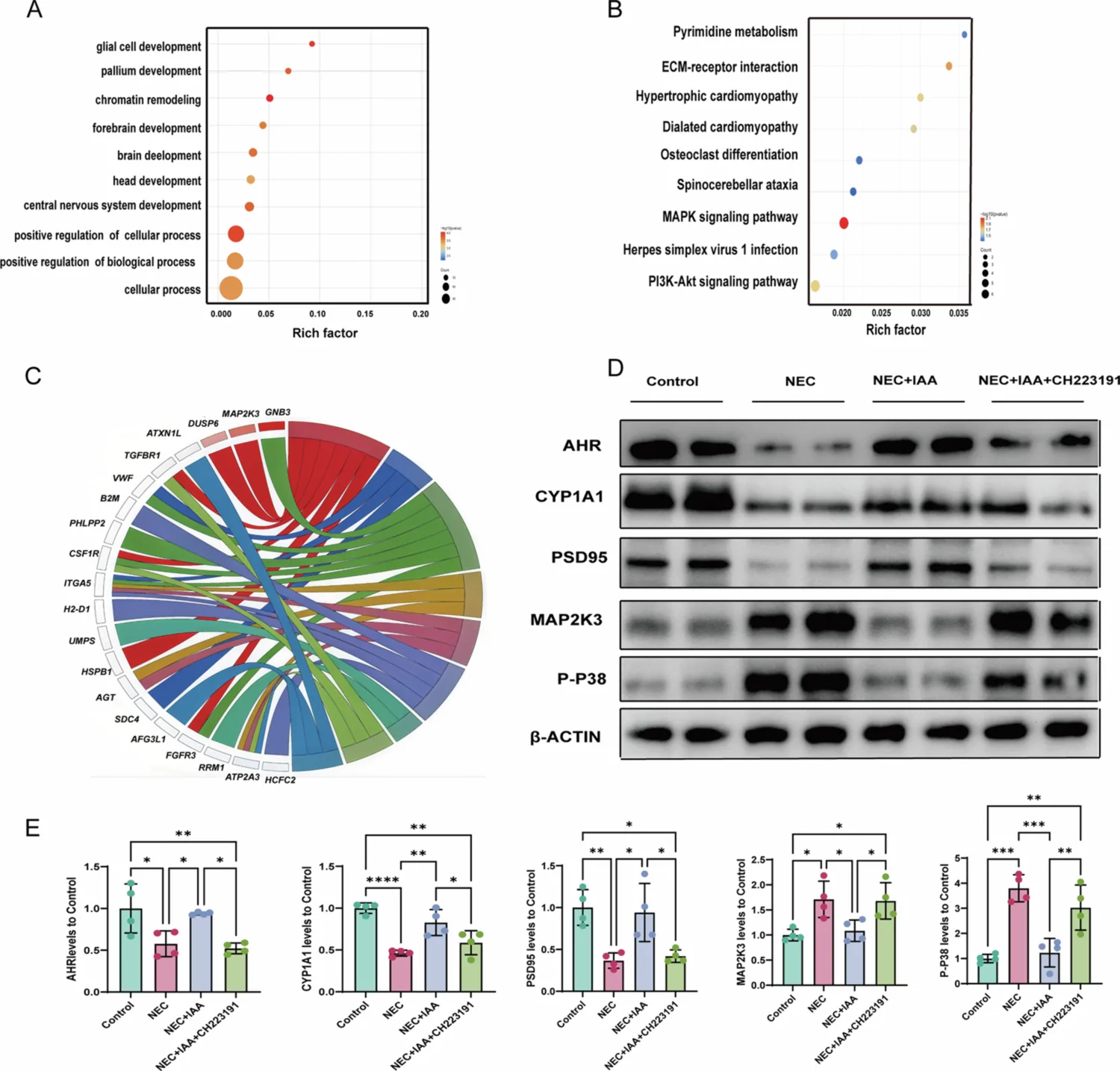
(A) GO biological process enrichment analysis of differentially expressed proteins. (B) KEGG pathway enrichment analysis. (C) Chord diagram showing the association between key differentially expressed proteins and enriched pathways. (D, E) Western blot analysis (D) and quantitative analysis (E) of the protein levels of AHR, CYP1A1, PSD95, MAP2K3, and phosphorylated p38 (*P*-P38) in the hippocampus of Control, NEC, NEC + IAA, and NEC + IAA + CH223191 mice. *β*-actin was used as a loading control. *N* = 4 per group. Data are presented as mean ± SD. *P < 0.05, **P < 0.01, ***P < 0.001, ****P < 0.0001, determined by one-way ANOVA followed by Tukey’s post-hoc test.

The role of the AHR/MAP2K3/p38 signaling axis was verified by Western blot analysis in the following four groups: control group, NEC group, NEC+IAA group, and NEC+IAA+CH223191 group (AHR inhibitor). Compared with the control group, the protein levels of AHR and its target gene CYP1A1 were significantly decreased in the NEC group. IAA treatment significantly upregulated the expression of AHR and CYP1A1, while CH223191 (AHR inhibitor) eliminated this effect (Figure 9D,E). At the same time, compared with the normal group, the expression levels of MAP2K3 and phosphorylated p38 (p-p38) were significantly increased in the NEC group, and IAA treatment reduced them, while CH223191 prevented this trend. The expression of the synaptic protein PSD95 was downregulated in NEC mice, and it was restored by IAA treatment, while it was reversed by CH223191. These results indicate that IAA activates AHR, inhibits the MAP2K3/p38 signaling pathway, thereby alleviating neuroinflammation and microglial activation, promoting the expression of synaptic proteins in NEC mice, and enhancing synaptic function.

## Discussion

Necrotizing enterocolitis (NEC) remains the most lethal gastrointestinal disorder in preterm infants, and survivors frequently suffer from irreversible long‑term cognitive impairment due to sustained gut‑derived neuroinflammation and synaptic damage, for which no targeted pharmacological agents are clinically available.^32^ The gut–brain axis has emerged as a core pharmacological target for neurological complications secondary to intestinal diseases,^33^ and β‑glucan, a natural prebiotic polysaccharide with well‑defined gut‑modulating and anti‑inflammatory properties, has been proposed to regulate neural function via microbial‑metabolic signaling. In the present study, we provide comprehensive preclinical pharmacological evidence that β‑glucan exerts neuroprotective effects against NEC‑induced long‑term cognitive impairment, and systematically delineate a sequential gut microbiota‑IAA‑AHR/MAP2K3/p38 signaling cascade underlying its therapeutic action.

Our behavioral results first confirmed that NEC mice exhibited pronounced severe cognitive deficits, as evidenced by reduced open‑arm exploration in the elevated plus maze, diminished central activity in the open-field test, impaired spatial memory in the Morris water maze, and defective novel object recognition. *β*-glucan effectively ameliorated the above phenotypic deficits, establishing a pharmacological basis for further mechanistic investigation. No significant difference was observed between low‑ and high‑dose β‑glucan in ameliorating cognitive impairment, which is closely associated with the physicochemical properties and targeting characteristics of β‑glucan. As a macromolecular polysaccharide, β‑glucan mainly exerts prebiotic effects locally in the intestine with extremely low absorption efficiency,^34^ so its therapeutic efficacy cannot be proportionally enhanced by dose escalation. Moreover, low‑dose β‑glucan has been sufficient to fully reverse gut microbiota dysbiosis and restore key metabolic responses, whereas high‑dose administration only increases its intestinal retention and provides limited additional benefits for strengthening gut–brain axis–mediated neuroprotection. Accordingly, we focused on gut microbiota, the principal target of *β*-glucan and an essential mediator of gut–brain axis homeostasis.^35^

Gut microbial dysbiosis is a well‑established pathological driver of NEC, characterized by depletion of beneficial commensals and overgrowth of pro‑inflammatory bacteria that trigger systemic and central inflammation.^36^ ^,^ ^37^ Our microbial profiling results demonstrated that NEC induced profound structural and compositional disturbance of the gut microbiota, with marked enrichment of Helicobacter, Klebsiella, and Desulfovibrionaceae, and depletion of Akkermania muciniphila, Lactobacillus, and Muribaculaceae. β‑glucan treatment robustly restored microbial homeostasis, shifting the community structure toward that of healthy controls as shown by PCA, and rebuilding a synergistic microbial network centered on Muribaculum and Bacteroides – key hub taxa maintaining intestinal and metabolic stability. Critically, fecal microbiota transplantation (FMT) from β‑glucan‑treated mice fully recapitulated the neuroprotective effects, including improved cognitive behavior, suppressed microglial activation, and restored synaptic protein expression. These results provide causal pharmacological evidence that the gut microbiota is not merely a biomarker but an indispensable functional mediator of β‑glucan‑induced neuroprotection, confirming that microbial remodeling is an upstream prerequisite for its therapeutic efficacy. All mice involved in the FMT‑related experiments in this study adopted an identical protocol for NEC modeling and antibiotic pretreatment. Therefore, the phenotypic comparison results between the NEC model group and the FMT intervention group receiving β‑glucan‑remodeled microbiota are reliable. Nevertheless, we cannot further distinguish the respective independent contributions of primary NEC‑induced injury, the intrinsic activity of β‑glucan, and the general effects of FMT. Accordingly, the protective effect shall not be solely attributed to β‑glucan per se or the general FMT effect.

To identify the key microbial effector metabolite, our metabolomic pathway enrichment analysis revealed that phenylalanine, tyrosine, and tryptophan biosynthesis was the most significantly modulated pathway, guiding us to focus on microbiota‑derived tryptophan metabolites. Tryptophan metabolism is an important metabolic pathway in the human body. As an essential amino acid, tryptophan can be converted into a variety of bioactive compounds through tryptophan metabolism.^38^ Since tryptophan metabolism is the most enriched pathway, we focused on tryptophan metabolites in this study to further explore their possible mechanisms. Tryptophan metabolism is considered a link between the gut microbiota and the brain.^39^ Numerous studies have shown that tryptophan metabolites play a key role in maintaining intestinal homeostasis and systemic immunity.^40^ In addition, tryptophan metabolites produced through various pathways can cross the blood-brain barrier, thus regulating the immune responses of astrocytes and microglia.^41^

Indole‑3‑acetic acid (IAA), a major tryptophan catabolite generated by commensal bacteria, is a selective endogenous agonist of the aryl hydrocarbon receptor (AHR)^42-44^ and plays a critical role in regulating neuroinflammation and synaptic homeostasis.^45^ ^,^ ^46^ Our metabolic results showed that IAA was drastically depleted in NEC mice and robustly restored by β‑glucan, consistent with the recovery of IAA‑producing commensals including Akkermania muciniphila^47^ and Lactobacillus.^48^ ^,^ ^49^

Previous studies have also shown that Akkermansia can increase the level of IAA in the intestine, which is consistent with the results of our study.^50^ ^,^ ^51^ Moreover, research has found that IAA can cross the blood-brain barrier and enter the central nervous system to exert neuroprotective effects.^52^

Pharmacological intervention further demonstrated that exogenous IAA supplementation mimicked the neuroprotective effects of β‑glucan, while the AHR antagonist CH223191 completely abolished these beneficial effects. These findings identify IAA as the key pharmacological effector metabolite and AHR as the critical upstream receptor transducing microbial signals to the brain, bridging gut microbial remodeling to central neuroprotection.^53^

AHR is a ligand‑activated nuclear transcription factor that resides in the cytoplasm in an inactive state under physiological conditions.^54^ Upon binding by its agonist IAA, AHR undergoes a conformational change, translocates from the cytoplasm to the nucleus, and heterodimerizes with the aryl hydrocarbon receptor nuclear translocator (ARNT).^55^ ^,^ ^56^ The activated AHR/ARNT complex specifically recognizes and binds to xenobiotic response elements (XRE) located in the promoter region of target genes, and exerts transcriptional repression by recruiting co‑repressor complexes (e.g., HDAC1/2) or competing with pro‑inflammatory transcription factors for DNA binding sites.^57^ ^,^ ^58^

Notably, MAP2K3 (also known as MKK3) is a direct transcriptional target gene negatively regulated by activated AHR.^59^ MAP2K3 is a specific mitogen‑activated protein kinase kinase that exclusively phosphorylates and activates the p38 MAPK signaling pathway, with no catalytic activity toward other MAPK family members.^60-62^ Hyperactivation of MAP2K3/p38 signaling is a core pathological driver of microglial overactivation, transcriptional upregulation of pro‑inflammatory cytokines, and synaptic structural damage in the hippocampus.^1^ ^,^ ^63-65^ Our hippocampal proteomic and Western blot results provided direct experimental validation for this regulatory axis: NEC significantly suppressed AHR transcriptional activity, leading to derepression of MAP2K3 transcription and marked upregulation of MAP2K3 protein expression, which in turn induced excessive p38 phosphorylation and sustained neuroinflammation. IAA treatment markedly activated AHR nuclear translocation and transcriptional activity, which directly transcriptionally inhibited MAP2K3 expression, reduced the phosphorylation of its downstream substrate p38, and blocked the pro‑inflammatory signaling cascade. In contrast, the AHR antagonist CH223191 reversed the inhibitory effect of IAA on MAP2K3/p38, confirming that AHR activation is an essential upstream molecular event for MAP2K3/p38 inhibition.

In accordance with the above signaling mechanism, the inhibition of MAP2K3/p38 by AHR mediated two core neuroprotective effects consistent with our results: first, it suppressed excessive microglial activation (reduced IBA1‑positive cells) and mitigated neuroinflammation, as reflected by decreased levels of pro‑inflammatory cytokines (IL‑1β, IL‑6, and TNF‑α) and increased expression of the anti‑inflammatory mediator IL‑22. Second, it preserved synaptic structural and functional integrity by upregulating PSD95 expression, thickening postsynaptic density, and increasing dendritic spine density in hippocampal CA1, CA2, CA3, and DG regions. These dual effects – resolving neuroinflammation and restoring synaptic plasticity – directly underlie the improvement of cognitive function and represent the terminal molecular pharmacological mechanism of β‑glucan and IAA.

## Conclusion

Collectively, β‑glucan reverses NEC‑induced gut microbial dysbiosis to restore the production of the microbial metabolite IAA; IAA acts as an endogenous AHR agonist to trigger AHR nuclear translocation and transcriptional activation; activated AHR directly transcriptionally represses MAP2K3 expression, thereby blocking p38 MAPK phosphorylation; this signaling cascade suppresses neuroinflammation and preserves synaptic structure and function, ultimately alleviating NEC‑induced long‑term cognitive impairment (Figure 10). This study expands the pharmacological landscape of β‑glucan beyond intestinal protection to neuroprotection via the gut–brain axis, and provides a preclinical rationale for developing microbiota‑targeted and IAA–AHR axis‑modulating therapies for NEC‑related neurocognitive complications.

**Figure10.**
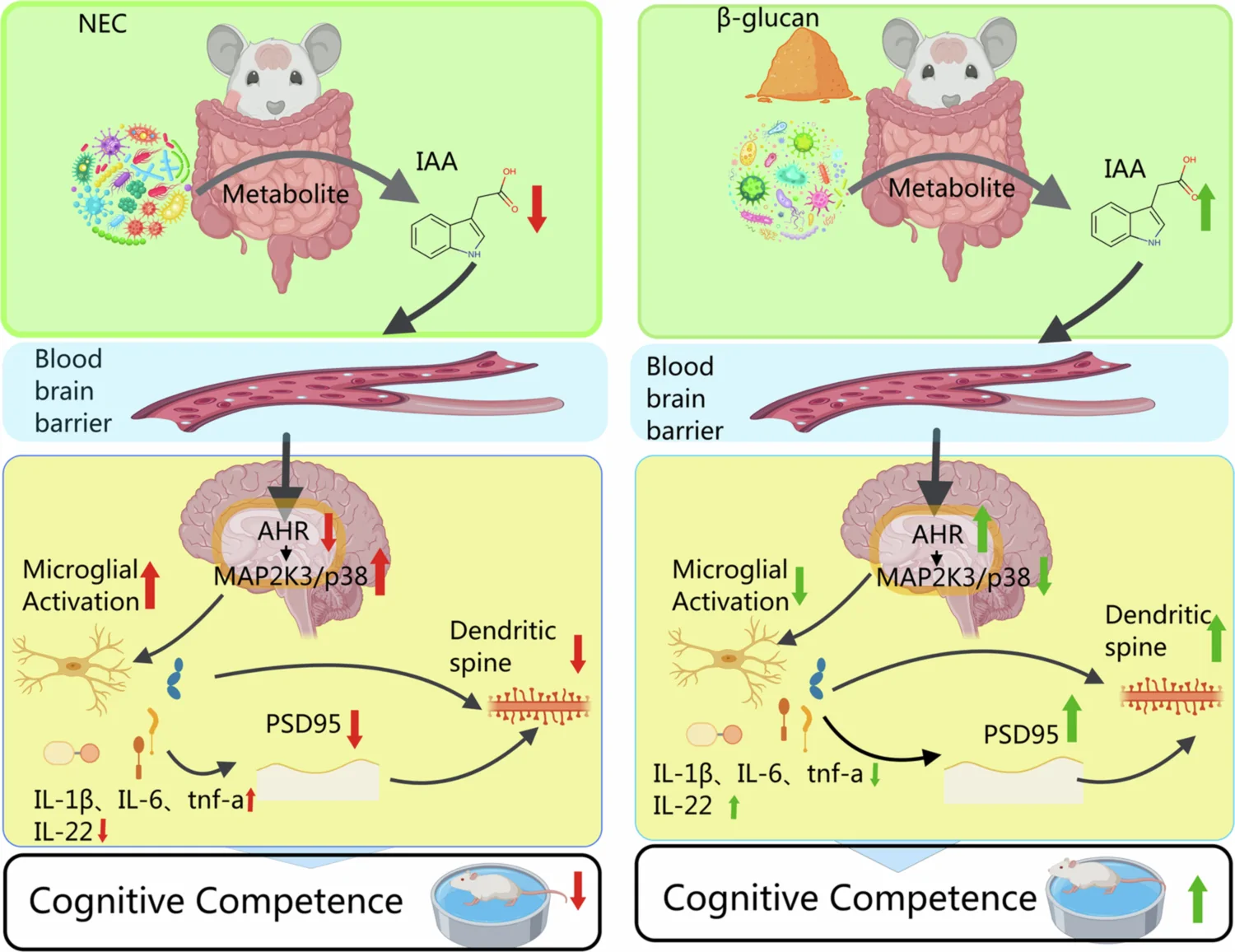
Mechanism diagram: *β*-glucan ameliorates NEC-related cognitive impairment through regulating the gut microbiota–tryptophan metabolism–IAA–AHR/MAP2K3/p38 gut–brain axis.

This study has several limitations that warrant future investigation. First, all experiments were conducted in a murine NEC model. In this model, we cannot distinguish the direct neurobiological consequences induced by hypoxia/hypothermia/maternal‑separation stress from the secondary neuroinflammation triggered by intestinal injury of necrotizing enterocolitis (NEC) in neonates. Dedicated experimental studies should be conducted subsequently with control groups subjected to stress only (with stress exposure but no intestinal injury from necrotizing enterocolitis), so as to dissect the independent effect of each stressor. Meanwhile, further pharmacological research in large‑animal models as well as clinical trials is required to verify the efficacy, safety, and optimal dosage of β‑glucan in preterm infants. Further pharmacological studies in large animals and clinical trials are required to validate the efficacy, safety, and optimal dosage of β‑glucan in preterm infants. Second, while IAA was identified as the primary effector metabolite, other tryptophan metabolites or microbial signals may act synergistically to enhance neuroprotection. Third, the precise binding site of AHR on the MAP2K3 promoter and the dynamic changes of co‑regulators require further verification by ChIP‑qPCR and luciferase reporter assays. Future research will focus on developing targeted IAA mimetics or selective AHR agonists, and optimizing β‑glucan formulations to advance translational applications for the prevention and treatment of NEC‑associated long‑term cognitive impairment.

## Supplementary Material

Supplementary Material New_Microsoft_Word_Document__1_.docx

Supplementary Table1.docx

## Data Availability

The gut metagenomics sequencing data in this study are available in the Sequence Read Archive (SRA) with project No. PRJNA1460552.
