# Supplementary material for: β-glucan mitigates necrotizing enterocolitis-induced long-term cognitive impairment by remodeling gut microbiota and restoring tryptophan metabolite IAA-mediated gut–brain signaling
Source: Gut Microbes. 2026 Nov 7;18(1):2756699. doi: 10.1080/19490976.2026.2756699 (PMC13757580; doi:10.1080/19490976.2026.2756699)
Supplement: Supplementary file 1 [file KGMI_A_2756699_SM4114.docx]

**Supplementary figure 1**

Quantification of key tryptophan metabolites in the control, NEC, and β-glucan groups.

Data are presented as mean ± SD. n = 8 per group. Statistical significance was determined by one-way ANOVA followed by Tukey’s post-hoc test.
