# Supplementary material for: β-glucan mitigates necrotizing enterocolitis-induced long-term cognitive impairment by remodeling gut microbiota and restoring tryptophan metabolite IAA-mediated gut–brain signaling
Source: Gut Microbes. 2026 Nov 7;18(1):2756699. doi: 10.1080/19490976.2026.2756699 (PMC13757580; doi:10.1080/19490976.2026.2756699)
Supplement: Supplementary file 2 [file KGMI_A_2756699_SM6317.docx]

|  | Group | Initial number of animals | Final valid sample size | Final valid sample /group |
| --- | --- | --- | --- | --- |
| Part 1 | Control | 20 | 15 | Behavioral Testing（n=7）  Targeted Metabolomics（n=8）  Gut metagenomics sequencing（n=8） |
|  | NEC | 20 | 15 |  |
|  | L-β glucan | 20 | 15 |  |
|  | H-β glucan | 20 | 15 |  |
| Part 2 | NEC | 40 | 33 | Behavioral Testing（n=7）  Immunofluorescence Staining（n=7）  Western Blot（n=4）  ELISA（n=4）  Transmission Electron Microscopy (n=7）  Golgi Staining（n=4） |
|  | NEC+FMT | 40 | 33 |  |
|  |  |  | 33 |  |
|  |  |  | 33 |  |
|  |  |  | 33 |  |
|  |  |  | 33 |  |
| Part 3 | Control | 45 | 38 | Behavioral Testing（n=7）  Immunofluorescence Staining（n=7）  Western Blot（n=4）  ELISA（n=4）  Transmission Electron Microscopy （n=7）  Golgi Staining（n=4）  Hippocampal proteomic analysis（n=5） |
|  | NEC | 45 | 38 |  |
|  | NEC+IAA | 45 | 38 |  |
|  | NEC+IAA  +CH223191 | 45 | 38 |  |
|  |  |  |  |  |
|  |  |  |  |  |
|  |  |  |  |  |

**Table1 Summary Table of Experimental Information**

**Note**: Actual survival numbers varied across groups; fixed sample sizes were selected from surviving animals to balance group size for statistical analysis, and surplus surviving mice were excluded from downstream experiments. Mortality mainly occurs during acute‑phase NEC induction.
